# In vivo imaging of the tumor and its associated microenvironment using combined CARS / 2-photon microscopy

**DOI:** 10.1080/21659087.2015.1055430

**Published:** 2015-06-08

**Authors:** Martin Lee, Andy Downes, You-Ying Chau, Bryan Serrels, Nick Hastie, Alistair Elfick, Valerie Brunton, Margaret Frame, Alan Serrels

**Affiliations:** 1 Edinburgh Cancer Research Center; Institute of Genetics and Molecular Medicine; University of Edinburgh; Edinburgh, United Kingdom; 2 School of Engineering; University of Edinburgh; Edinburgh, United Kingdom; 3 Medical Research Council Human Genetics Unit; Institute of Genetics and Molecular Medicine; University of Edinburgh; Edinburgh, United Kingdom

**Keywords:** CARS, Cancer, Coherent Raman microscopy, Harmonic generation, Invasion, Intra-vital microscopy, Multi-photon, Optical Window, Squamous Cell Carcinoma, SHG, Tumor microenvironment

## Abstract

The use of confocal and multi-photon microscopy for intra-vital cancer imaging has impacted on our understanding of cancer cell behavior and interaction with the surrounding tumor microenvironment *in viv*o. However, many studies to-date rely on the use fluorescent dyes or genetically encoded probes that enable visualization of a structure or cell population of interest, but do not illuminate the complexity of the surrounding tumor microenvironment. Here, we show that multi-modal microscopy combining 2-photon fluorescence with CARS can begin to address this deficit, enabling detailed imaging of the tumor niche without the need for additional labeling. This can be performed on live tumor-bearing animals through optical observation windows, permitting real-time and longitudinal imaging of dynamic processes within the tumor niche.

## Introduction

The dependency on complex tumor / host interactions for cancer progression has driven the need to exploit advanced imaging technologies such as confocal and multi-photon laser scanning microscopy (MPM) to image deep in tumor tissue in living animals. The increasing spatial and temporal resolution of these microscopy techniques, together with the improved imaging depth offered by multi-photon excitation, has led to studies of exquisite detail at both the cellular^1-5^ and subcellular level, including the application of advanced imaging techniques such as Fluorescence Recovery After Photo-bleaching (FRAP),^6,3^ Photo-activation, ^6^ Photo-switching, ^3^ and Fluorescence Lifetime Imaging (FLIM)^7-10^ within the complex in vivo environment. However, to-date the most commonly used techniques require the expression of a fluorescent marker in order to generate sufficient image contrast. Such markers are usually highly specific and only enable visualization of a single population of cells or a single type of tissue structure e.g. blood vessels, and thus provide limited contextual information.

Increasingly 2-photon fluorescence (2-PEF) is being combined with ‘label-free’ non-linear optical methodologies, including Second and Third Harmonic Generation (SHG and THG, respectively) for intra-vital cancer imaging. This enables visualization of a range of structures deep within tumor tissue, including fibrillar collagen,^1,11^ nerves, muscle, adipose cells, and blood vessels,^2,12^ providing greater detail within the image without the need for further addition of exogenous labels. While SHG is relatively specific for imaging collagen and striated myofibers, THG has offered a more comprehensive view of surrounding tissue structures, although there remains some issues with the use of high powered femtosecond pulses which may cause damage, particularly when continuously imaging in the same plane. ^2,13^ None-the-less, this move toward a combinatorial approach of ‘label-dependent’ and ‘label-free’ imaging is attractive as it provides specificity together with a more general overview of tissue architecture.

Since its resurrection in 1999 by the Xie group at Harvard, Coherent Anti-Stokes Raman Scattering microscopy (CARS) has emerged as a promising ‘label-free’ imaging modality that can be applied to biomedical samples. CARS is a form of fast-acquisition Raman microscopy that generates image contrast using the Raman active vibrational frequency of given chemical bonds, and can be tuned over the Raman spectrum to generate image contrast from a wide variety of chemical bonds present within biological samples. The CARS process is described in more detail elsewhere.^14,15^ In the cancer setting, CARS has been used to identify changes in lipids within the tumor microenvironment that occur when on a high-fat diet,^16^ and to differentiate between normal and malignant brain tissue.^17^ Furthermore, the few live animal studies performed to-date have focused on imaging lipids in the skin, ^18^ small intestine,^19^ and circulating cancer cells,^20^ and to assess animal myelin histomorphometry.^21^ Thus, further investigation is still required to fully exploit the potential of CARS as an intra-vital imaging tool, both alone and in combination with other imaging techniques. Here, using a custom-designed multi-modal CARS imaging system, we have investigated the utility of CARS as a pre-clinical cancer-imaging microscopic tool. When combined with 2-PEF and SHG, we show that CARS permits direct visualization of the complex tumor microenvironment and the interaction between tumor and host, without the addition of label, and at laser powers that maintain good sample viability.

## Results

### Chemically selective imaging

**Figure 1.**
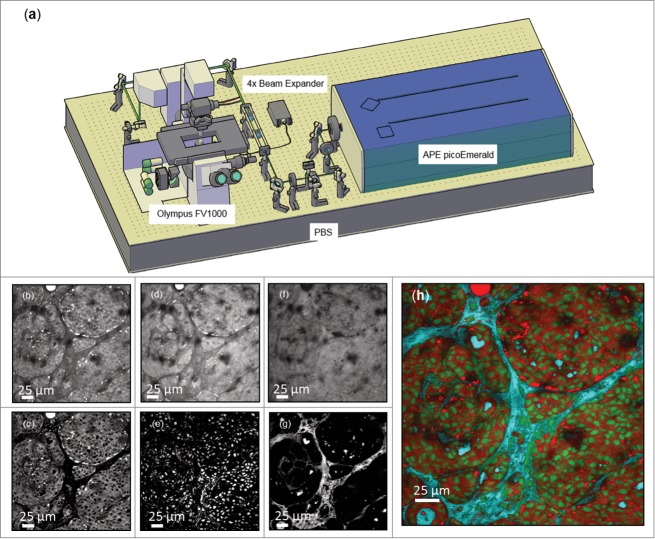
Chemically selective imaging of tumor histopathology in MMTV-PyMT mammary tumors. (**a**) Schematic of the custom designed microscope used throughout the study – see methods section for further details. Images acquired of tumor bearing mammary fat pads from the MMTV-PyMT mouse at (**b**) 2,845 cm^−1^, (**d**) 2,930 cm^−1^ and (**f**) 3,030 cm^−1^ highlighting tissue features visible at different CARS frequencies. Each image contains contributions from OH, CH_3_ and CH_2_ stretches in different proportions. To highlight the tissue features corresponding to the 3 chemical stretches (termed OH – water, CH_3_ – nucleus and CH_2_ – cell cytoplasm and lipid droplets) the images were subtracted from one another then manually thresholded. The OH stretch image (**g**) was created from 3,030 cm^−1^ – 2,930 cm^−1^ images, the CH_2_ stretch image (**c**) was from 2,845 cm^−1^ – 3,030 cm^−1^ images, while the CH_3_ stretch image (**e**) is created from subtracting the generated OH and CH_2_ images from the 2,930cm^−1^ image. (**h**) Combined false color image appears as a pseudo H & E stain, highlighting nuclei (CH_3_) in green, cytoplasm (CH_2_) in red and water (OH) in cyan.

Acquisition of spectral information using vibrational imaging techniques including CARS, stimulated Raman scattering (SRS), and nonlinear interferometric vibrational imaging (NIVI), has been reported to enable identification of cells with different morphological features ^18,22^ and to aid discrimination between normal and malignant tissue. ^22–24^ Using a custom designed 2-photon / CARS microscope we examined tumor bearing mammary fat pads immediately following surgically removal from MMTV-PyMT mice ^25^ and acquired images at 3 different CARS frequencies; 2,845 cm^−1^ which has been associated with the lipid asymmetric CH_2_ stretch, 2,930 cm^−1^ which has been associated with the protein rich CH_3_ stretch, ^17^ and 3,030 cm^−1^ which has been associated with the water OH stretch. ^22,26^ Images acquired at 2,845cm^−1^ (**Fig. 1b**) showed strong contrast from lipids and the cytoplasmic component of cells, with nuclei yielding a weaker signal. The area between cell clusters exhibited little contrast. In comparison, images acquired at 2,930cm^−1^ (**Fig. 1d**) showed good lipid and cytoplasmic contrast, but also exhibited a prominent nuclear signal. Images acquired at 3,030 cm^−1^ (**Fig. 1f**) appeared as a negative of that acquired at 2,845 cm^−1^, highlighting the interstitial fluid filled space surrounding the clusters of tumor tissue. Processing of this spectral image series using image-based subtraction and thresholding (described in detail in Methods) resulted in a combined image akin to a pseudo H & E stain (**Fig 1h**), highlighting nuclei, cell bodies, and tissue architecture, similar to that reported previously using SRS. ^24^ Thus, chemically selective imaging using CARS may enable label-free histopathology of cancerous tissue, and could provide a rapid intraoperative assessment of breast pathologies in a similar manner to that proposed using a combination of non-linear microscopy and a fluorescent nuclear stain. ^27^ Furthermore, such an approach should facilitate label-free longitudinal imaging of tumor development in mouse models of cancer, especially when combined with the use of optical imaging windows.

### Imaging cancer in vivo and the tumor microenvironment using multi-modal imaging

**Figure 2.**
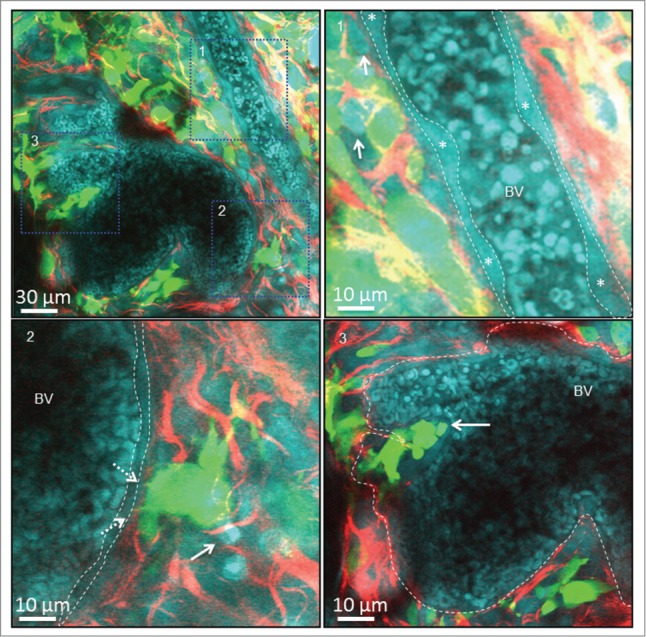
Multi-modal imaging of cancer. *Top Left –* Representative image from a z-stack taken 35 μm below the tumor surface showing GFP-labeled SCC cancer cells (Green), collagen matrix imaged using SHG (red), and a range of cell types including red blood cells (RBC), endothelial cells, and tumor infiltrating immune and stromal cells imaged using CARS (Cyan). *Top right -* Small region from the left image showing RBCs, endothelial cells lining a blood vessel (each cell body is marked with *) and infiltrating host cells (solid arrow). *Bottom left* –Small region from the top left image showing a tumor cell pushing out invadopodia or fillopodia (dashed arrow) through the perivascular collagen toward the endothelial lining (dashed line) of the adjacent blood vessel. This cell appears to be in contact with host cells (solid arrow). *Bottom right –* Small region from the top left image showing a group of cancer cells (solid arrow) that have invaded through the perivascular collagen (boundary delineated with dashed line) and are pushing on the endothelial cell wall of a blood vessel.

To date most cancer-based CARS microscopy studies have focused on its potential as a diagnostic tool (as described above), with the aim of label-free identification of tumor tissue and malignant cancer cells. ^23,24,28^ However, very little has been done to investigate the potential utility of CARS to image the cancer cells and the relationship of these to their immediate environment. We therefore examined tumor tissue following surgical resection from the mouse by combining CARS, 2-PEF and SHG. Using tumors established from a GFP-expressing mouse Squamous Carcinoma Cell line (SCC), we simultaneously acquired CARS images at 2,930 cm^−1^, together with 2-photon excited GFP fluorescence and SHG arising from the collagen matrix (**Fig. 2** and **Supplementary Movie 1**). The images highlighted a number of interesting features that could be discerned by the co-generation of a CARS image. Firstly, Red Blood Cells (RBC) within tumor infiltrating blood vessels and the endothelial cells layering the wall of the vasculature were readily visualized (**Fig. 2** top right panel, outlined with dashed white line). Moreover, we could identify individual endothelial cell bodies lining the vasculature (**Fig. 2** top right panel, asterisk), enabling us to establish that endothelial cells were present and that the endothelial wall of the vasculature was intact. Secondly, cancer cells can be seen pushing out small protrusions, potentially what others have called invadopodia or filopodia, through the perivascular collagen to contact endothelial cells in what could represent the early stages of intravasation (**Fig. 2** bottom left panel, broken arrows). Interestingly, the CARS image also showed that these cells are in contact with a host cell, which would support the role of tumor/host interaction during intravasation (solid arrow). Thirdly, using SHG we observed groups of cancer cells invading through the perivascular collagen, and pushing on the endothelial cell wall creating an invagination into the blood vessel (**Fig. 2** bottom right panel, solid arrow). This may represent further stages of intravasation post-degradation of perivascular collagen, but prior to breaching of the endothelial lining of the blood vessel. As shown here, the combination of CARS, 2-PEF, and SHG can provide excellent resolution of the relationship between tumor cells and features of their environment, and this may help to define the process of intravasation over time.

**Figure 3.**
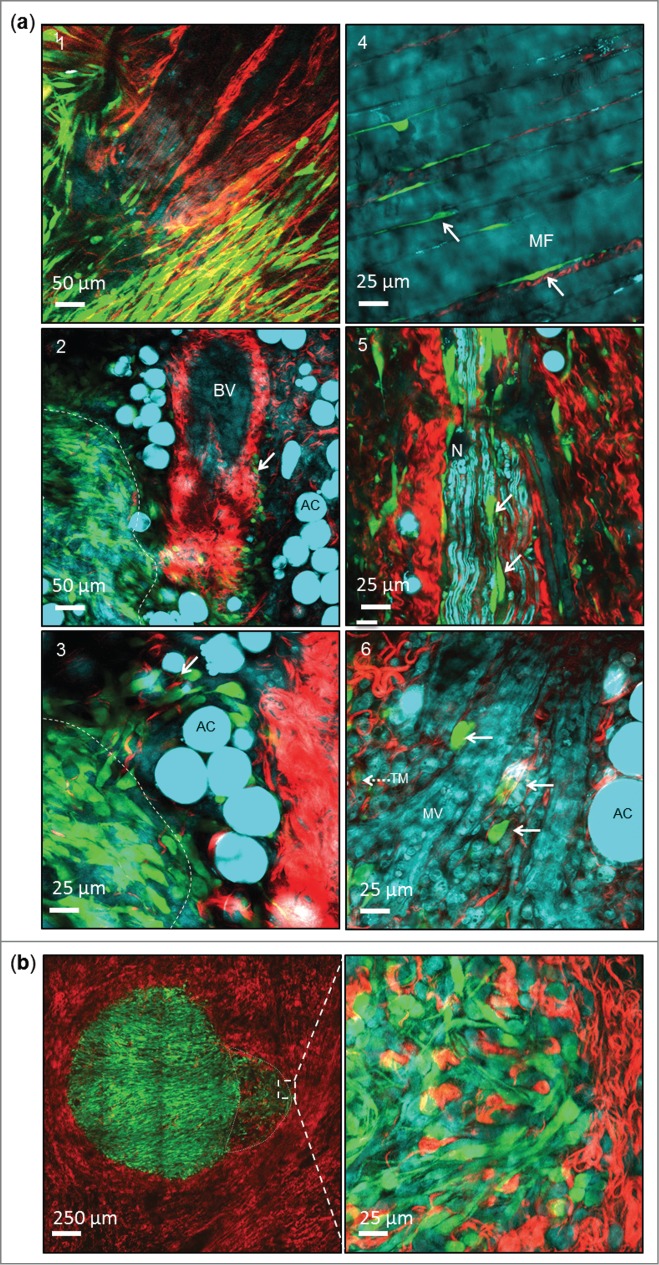
Multi-modal imaging of cancer invasive tracks. (**a**) Representative images showing how a combination of SHG (Red) and CARS (Cyan) can be used to identify a range of invasive tracks used by cancer cells (Green). Tumor margin, dashed line; invading cells, solid arrow; BV, blood vessel; AC, adipose cell; TM, tumor margin; MF, muscle fiber; N, nerve. (**b**) Representative image showing tumor / host interaction at the invading margin of a tumor. GFP-labeled SCC cancer cells (Green) are seen invading out from the tumor margin through the collagen matrix (SHG - Red). These invading tumor cells are accompanied by an intense host cell component imaged using CARS tuned to 2,845 cm^−1^ (Cyan). The invading margin is delineated with a gray dashed line and the area magnified in the right image is marked by a white box on the left image.

Cancer cells are thought to use a variety of structures as tracks along which to invade, including collagen fibers, adipose cells, nerves, blood vessels, and muscle fibers.^2,12^ Using THG, Friedl and colleagues were able to visualize these components of the microenvironment.^2^ We therefore addressed whether CARS could also enable imaging of such structures, but providing the advantage of using lower laser powers that do not cause visible photo-damage when continuously scanned (**Fig. S1** and **Supplementary Movie 7**). Using GFP-labeled SCC tumors immediately following surgical resection from the mouse, we acquired CARS, 2-PEF, and SHG images from a variety of regions at the tumor margins (**Fig. 3a**). Using SHG we were able to visualize cancer cells aligned along the collagen matrix (**Fig. 3a** image 1), and invading along the perivascular collagen of a large blood vessel that was identified by CARS (**Fig. 3a** image 2, solid arrow). In addition, using CARS we could also see cancer cells interacting with adipose cells and local sites of invasion around clusters of adipose cells (**Fig. 3a** image 3, solid arrow), muscle fibers (Fig. 3**a** image 4, solid arrows), nerve cells (**Fig. 3a** image 5, solid arrows), and microvascular structures within the skin (**Fig. 3a** image 6, solid arrows).

**Figure 4.**
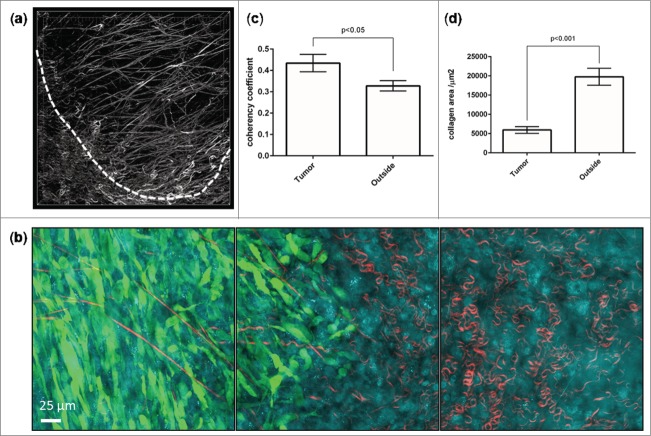
Quantifying collagen changes. (**a**) Representative render showing the collagen present at the border of an SCC tumor (indicated by dashed line). Z-stack taken between 30 and 67 μm below the tumor capsule. (**b**) Representative images taken from a 3 image series scanned across the border of a tumor with the central image aligned perpendicular and central to the tumor border. Collagen coherency and amount was calculated from the left and right images via the OrientationJ plugin for ImageJ and thresholded area respectively. (**c and d**) Collagen coherency and abundance (mean ± SEM) in tumor and outside. n = 22, paired t-test.

It has also previously been reported that host cells, such as macrophages and stromal fibroblasts, can assist tumor cell invasion and intravasation by a variety of mechanisms.^4,5,29^ Using multi-site image acquisition of a 5 × 5 grid and subsequent image tiling, we reconstructed a GFP-labeled SCC tumor within the skin and identified a tumor edge with cells invading out into the surrounding collagen (**Fig. 3b**, left panel, **Supplementary Movie 2**). CARS imaging at the invasive edge revealed an area of intense tumor / host interaction, supporting the recognized role that host cells play in assisting this process. At this point we do not know the identity of the host cell/s identified using CARS. It has been shown that tumor cells are extensively involved in re-modeling the extracellular matrix.^30^ We observed marked changes in the collagen abundance and orientation within the tumor (**Fig. 4a**) with collagen appearing as less dense, straighter and more aligned than those found outside of the tumor boundary. To quantify this result sequential images were taken at tumor borders (**Fig. 4b**) so that the center image contained the border of the tumor and the collagen content from the adjacent images represented tumor and outside. The collagen content of the images were then measured for coherency, which represents the propensity for collagen fibers to be aligned in the same orientation, ^31^ and abundance. Collagen fibers within the tumor were found to be significantly less abundant and more orientated (**Fig. 4c and d**) than those found outside the tumor suggesting that tumor cells may be responsible for orientating and degrading collagen fibers.

**Figure 5.**
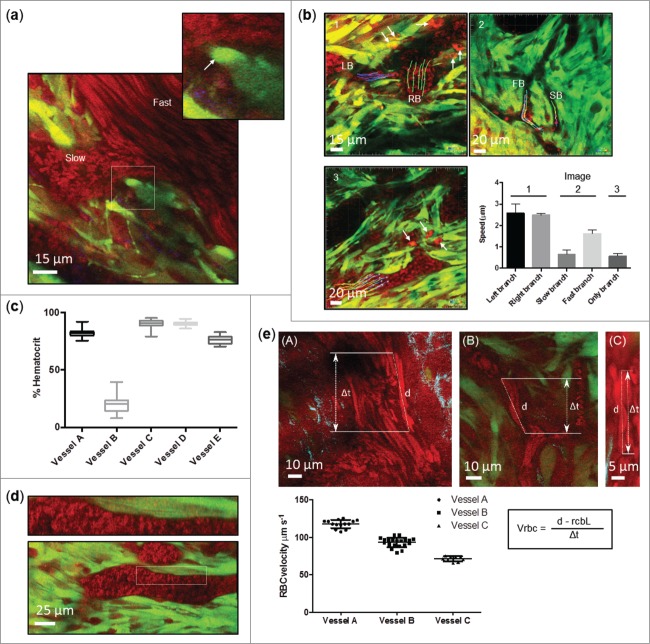
Quantifying tumor blood flow. (**a**) Representative image showing heterogeneity in blood flow rate within tumors. A cancer cell can be seen in close contact with Red Blood Cells (RBC), area magnified is indicated with dashed box and potentially intravasating cell marked with solid arrow. (**b**) Representative images taken from 3 separate time-lapse series acquired through dorsal skinfold windows showing label-free tracking of RBC flow within tumor vasculature (multi-colored lines). Lipid rich perivascular host cells (solid arrow), possibly macrophages, are evident. *Right –* Graph showing quantification of 5 slow moving vessels showing heterogeneity of RBC flow rate between different vessels. Only branch n =14; Fast branch, Left branch, Right branch n = 5; Slow branch n = 3. (**c**) Hematocrit quantification of 5 slow moving vessels. Each image from a 40 image time-series was quantified and standard error of the mean calculated. Graph shows median, upper and lower quartile, and minimum / maximum values. (**d**) Representative image showing RBCs in close contact with tumor cells in what may represent vascular mimicry. Upper panel is magnified area marked by dashed box in lower panel. (**e**) Representative images taken from 3 separate time-series’ acquired through dorsal skinfold windows showing label-free quantification of fast-flowing RBCs within tumor associated blood vessels. *Bottom –* Graph showing quantification of RBC velocity within each of the 3 vessels. A minimum of 8 blood cells were tracked. *Right bottom –* formula used to calculate RBC velocity (Vrcb), d = distance traveled during scan, t = time, rcbL = average red blood cell diameter. Image acquisition for all images was performed using the CARS stretch at 2,930 cm^−1^ (Red) and acquired alongside GFP-labeled SCC cancer cells (Green) and SHG (cyan).

We next addressed whether CARS could be applied in vivo using dorsal skinfold optical observation windows, enabling dynamic measurements at moderate laser powers that do not compromise tissue viability. We found that image quality was slightly lessened when using windows, although features such as endothelial cells remained visible (**Supplementary Video 4**). Movement of the tissue was kept to a minimum as the window was clamped in place, but breathing and other mouse movement can cause flickering artifacts across sections of the image. We focused on blood flow as an example of a highly dynamic process occurring within tumors, and asked whether CARS could provide a quantitative measure of relative blood flow, so permitting sub-classification of blood vessels. Time-lapse imaging of blood flow within GFP-labeled SCC tumors established under optical windows revealed considerable heterogeneity in the rate of blood flow, with vessels displaying different blood flow speeds branching from each other (**Fig. 5a**). Using particle tracking in silico, we determined the velocity of un-labeled individual RBCs flowing through a variety of vessels (**Fig. 5b** and **Supplementary Movies 4 - 6**), and using image thresholding we calculated the area occupied by RBCs over the total area of the blood vessel as a measure of hematocrit (the percentage of RBCs in the blood) (**Fig. 5c**). Both RBC velocity and hematocrit have been reported to affect tissue oxygenation levels, and to affect each other with a consequent impact on oxygenation^32^. Thus, it may be possible to infer the chances of a given tumor region being subject to low oxygen levels and hence its degree of hypoxia. In this regard, one could also analyze responses to vascular modifying anti-cancer agents. It was also notable that RBCs in some vessels were in very close contact (less than 1 μm) with cancer cells, which may suggest an absence of an endothelial vessel wall (**Fig. 5d**), although the visibility of these cells are affected by the contrast of the surrounding tissue, and the faster acquisition times used in live imaging. For blood vessels in which RBC velocity was too fast for particle tracking, i.e. those in which the blood flow was faster than the acquisition frame rate, we adopted an alternative method for image acquisition and analysis originally proposed by Kamoun *et al*, termed Relative Velocity Field Scanning (RVFS).^33^ This technique is based on the use of slow scanning speeds to create distorted images of RBCs and the residence time and distance traveled used to calculate velocity (**Fig. 5e**). Application of this approach using CARS images successfully enabled the quantification of RBC velocity where single cell RBC tracking was not possible, and further highlighted the heterogeneity of blood flow rate within tumor vasculature. Therefore, CARS provides a visualization and analysis tool for blood vessels, and enables their sub-categorization based on flow rate and RBC content without the use of any label. When combined with 2-PEF this analysis may facilitate correlation between blood flow parameters and localized tumor cell characteristics. It represents a viable label-free approach to complement other techniques that use the standard vascular contrast agents, such as fluorescently conjugated BSA, dextrans, or quantum dots. While these label vascular structures, to our knowledge they do not enable direct visualization of blood cells although it may be possible to infer these findings from the negative contrast of blood cells against a labeled vascular agent.

## Discussion

We have found that simultaneous acquisition of 2-PEF, SHG, and CARS imaging modalities can enhance detailed studies within un-processed tissue ex vivo and in tumor bearing live animals, and at laser powers that do not cause visible photo-damage. One strength of CARS as a pre-clinical imaging tool is to enable detailed description of ‘in tissue’ environments over time, and the relationship between cancer cells and their niche, particularly when used in combination with 2-PEF and SHG. Together these can provide exquisite detail of tissue structures, blood flow, vascular integrity, tumor / host interaction, and tumor associated processes that are visibly linked to invasion and intravasation.

Third Harmonic Generation is a valuable technique to identify tissue structures in tumor environments, and has been instrumental in identifying that cancer cells exploit a variety of structures such as adipose cells, nerves, and muscle as tracks along which to invade and migrate^2^. However, there are some issues with timelapse and continuously imaging the same area of tissue with *Drosophila* embryo survival dropping when the illumination time was above 20% of the total imaging time and blood flow monitored in mouse dorsal skin fold chambers ceasing after 60 seconds of continuous imaging.^2,13^ Here, we have shown that CARS, using a picosecond pulsed laser source, can permit imaging of a similar variety of structures within tissue (**Fig. 3**). CARS is also able to continuously image the same region without visible photodamage to track dynamic changes. Indeed, 300 frames of continuous imaging did not show any signs of visible photo-damage, suggesting that the image acquisition parameters being applied are conducive to long-term imaging deep within tissue (**Fig. S1** and **Supplementary Movie 7**). These benefits are further enhanced by the added flexibility of being tunable to produce images from across the Raman spectrum, yielding detailed morphological and structural information similar to that attained from haematoxylin and eosin staining routinely used in histological examination of fixed tissue. We conclude that multi-modal CARS / 2-photon imaging represents a very promising method for imaging structure / function relationships within cancer tissues and at exposure to modest laser power.

The application of multi-modal CARS imaging to the real-time study of cancer in vivo has the potential to impact in a number of important areas that remain poorly understood. Using this approach we have observed cancer cells projecting structures, akin to what others have termed invadopodia or filopodia, through the perivascular collagen network to contact the endothelial cells lining the vasculature. These invasion-associated structures are known to be rich in matrix metalloproteinase activity, and drive local degradation of the extracellular matrix (ECM).^34^ Interaction with tumor infiltrating host cells may play an important role in promoting this behavior, and multi-modal imaging enables the real-time visualization of such complex 4-way interactions between cancer cells, the ECM, endothelial cells, and tumor infiltrating host immune cells. Thus the temporal kinetics of such complex interactions could be monitored and help inform on the dynamic nature of tumor / host interactions that underpin processes including intravasation and extravasation.

Tumor vasculature plays a vital role in not only sustaining tumor growth, but also in enabling tumor cell dissemination and ultimately metastasis. Properties including blood flow rate and hematocrit will likely have effects on local tumor cell behavior, through for example influencing local oxygenation or through increased shear forces affecting survival of intravasating cancer cells. Imaging these properties in conjunction with local tumor cell behavior may help us understand the influence of blood flow characteristics and their local impact on cancer cells and even activity of cancer driver pathways. Indeed, measurements of cancer driver pathway activation, such as Src,^8^ RhoA ^9^ and Rac,^7^ have recently been reported using in vivo FLIM imaging of FRET (Fluorescence Resonance Energy Transfer) biosensors, and others have shown that multi-modal CARS 2-photon imaging can be combined with FLIM.^35^ Therefore interrogation of the relationship between blood flow characteristics and intracellular signaling in cancer cells may be realistically achievable using a combination of these technologies integrated onto a single imaging system. In addition to quantification of RBC velocity and number, CARS also facilitates visualization of endothelial cells lining the blood vessels and visual inspection of the integrity of the endothelial lining of the blood vessel. In combination with 2-photon fluorescence to image fluorescently labeled cancer cells, this could represent a valuable approach for real-time identification of ruptured vascular walls during the process of intravasation, or even the identification of vascular mimicry.

In summary, here we provide evidence that CARS has enormous potential when combined with 2-photon microscopy in imaging the internal environment in tissue and tumors, including in living animals. In the future, an exciting possibility is that the successes of Raman based techniques in identifying immune cell types,^36,37^ differences between normal, benign and malignant tissues,^38-40^ and the discrimination between drug sensitive and drug resistant tumors,^41^ may be transferable to coherent Raman imaging by further developing and exploiting the spectroscopic data generated while imaging.
