# Supplementary material for: In vivo imaging of the tumor and its associated microenvironment using combined CARS / 2-photon microscopy
Source: Intravital. 2015 Jun 8;4(1):e1055430. doi: 10.1080/21659087.2015.1055430 (PMC5226011; doi:10.1080/21659087.2015.1055430)
Supplement: Supplementary_Materials.zip [file kinv-04-01-1055430-s007.zip › 1055430_Supplementary Materials.docx]

**Supplementary Materials:**

**Materials and Methods:**

**Microscope Design and Construction**

The APE picoEMERALD laser system was used to provide two spatially and temporally aligned laser beams, the pump (tunable between 720-980 nm, 5-6 ps, 80 MHz repetition) and the Stokes (fixed 1064 nm, 7 ps, 80MHz repetition rate), that were aligned into an Olympus FV1000MPE (with mini-scanhead) confocal scanning microscope. The laser output was aligned through a fast shutter (Uniblitz VS25) that was synchronized with the scanning unit of the confocal microscope using a Uniblitz VCM-D1 single channel shutter driver. The laser beam was then reflected at 90° using a dielectric mirror (DeM) and aligned through a half-wave plate onto a second 90° DeM. This half-wave plate enabled rotation of the polarization of the laser light so that when it is passed through a polarizing beam-splitter cube (PBS), which is located after the second DeM, it is either reflected at a 90° angle onto a beam block or transmitted through the PBS onto the next optical component. Although laser power can be controlled within the laser box, this setup enables manual control of laser power within the light path and provides an additional over-ride and safety feature. The laser light was then passed through a half-wave and onto a third 90° DeM that directs the beam through a beam expander. The beam expanding setup used was designed to enable maximum flexibility in objective choice, and therefore had two magnetically mounted flip-in lenses at pre-defined distances (the sum of the two focal lengths) from the first fixed lens within the setup. This enables the user to change between 2x and 4x beam expansion, generating a choice of two beam diameters compatible with optimal performance of most Olympus objective lenses. The expanded beam was then reflected at a 90° angle by a fourth DeM and aligned into the confocal scanhead using two further DeMs as steering mirrors. The specifications and product codes for all optical components used in this light path setup are detailed in supplementary table 1.

Within the FV1000 confocal microscope the laser beam was further expanded to ensure the 15.2 mm back aperture of a 25× 1.05 NA water (XL Plan, Olympus) objective lens was correctly filled. Laser light passing through the microscope was directed into the objective lens using a short pass 690 nm dichroic mirror (Olympus) mounted in the filter turret. Back-scattered emission was filtered using the same dichroic mirror, and then reflected through an IR cut filter (ET910SP, Chroma) using an internal mirror. Fluorescence, SHG and CARS signals were detected simultaneously using a filter cube for spectral separation of emission signals onto one of four available non-descanned PMTs. The filter details were as follows: dichroic mirrors: FF01-552-Di02, FF705-Di01 and LM01-466, filters: SHG/blue FF01-440/40; green FF01-510-84; CARS/red FF01-609/181 (Semrock). The pump laser was tuned to 805.0 nm for CARS images taken at 3,030 cm^-1^, 812.2 nm for CARS images taken at 2,930cm^-1^ and 816.8 nm for CARS images at 2,845 cm^-1^.

**Ex vivo Tissue Imaging**

Mice were euthanized using cervical dislocation and tissue removed immediately for imaging. To enable imaging the tissue was placed directly onto a glass bottomed 30 mm tissue culture dish (World Precision Instruments) and PBS applied to ensure tissue remained hydrated over the imaging period. Images were captured using the custom-built microscope setup detailed above equipped with an Olympus XLPlan N 25x 1.05 N.A. water immersion lens. At-sample laser powers applied were in the range of 30 – 60 mW for both the pump and Stokes beams, with typical pixel dwell times of 10 𝜇s used with images gathered every 2.6 seconds. Emission signals were spectrally separated using the filter sets detailed in the ‘Microscope Design and Construction’ section.

**Collagen Quantification**

A series of three adjacent images were taken using the automated stage of the FV1000 microscope with the center image bisecting the tumor boundary vertically between 30-50 𝜇m below the tumor capsule. Adjacent images were termed tumor or outside depending on the tumor orientation. 12 tumors were examined between 2 and 7 days post injection, up to 3 sets of images were recorded at different boundary regions for each tumor for a total of 22 images of each condition. Collagen coherency was measured using the measure function of the OrientationJ plugin for ImageJ ^42^ with sigma set to 10. Area covered was measured by thresholding collagen from the background and using the measure function of ImageJ. Statistics were calculated using paired, two tailed t-test.

**Image processing for generation of a pseudo H & E stain**

Using images acquired serially from the same tissue region at 3,030 cm^-1^, 2,930 cm^-1^, and 2,845 cm^-1^ a composite image was generated as follows: the OH stretch image was created by subtracting the 2,930 cm^-1^ image from the 3,030 cm^-1^ image, the CH_2_ stretch image was created by subtracting the 3,030 cm^-1^ image from the 2,845 cm^‑1^ image, and the CH_3_ stretch image was created by subtracting the new OH and CH_2_ images from the 2,930cm^-1^ image. Images were thresholded manually to adjust contrast and brightness. The modified images were combined into a single false coloured image highlighting nuclei (CH_3_) in green, cytoplasm (CH­_2_) in red and water (OH) in cyan. All image processing was performed using ImageJ.

**Surgical implantation of optical window chambers**

Optical window chambers were implanted into CD-1 nude mice as previously described. ^3^ All animal work was carried out in compliance with UK Home Office guidelines. Briefly, the dorsal skin was pinched down the mid-line of the back using the symmetry of the feeding and draining vessels of the back as guide, and sutured onto a template to suspend the skin. A circle of skin was removed from one side and a 2 millimeter (mm) biopsy punch used to create the required screw holes. The titanium frame of the window was then fitted to either side of the skin and secured using screwing nuts, taking care not to occlude any blood vessels. The window was then further secured using five sutures and the clamp removed. A small tumor fragment (approximately 1 mm in diameter), or a preparation of 1 x 10^6^ cells in 10 μl Phosphate Buffered Saline (PBS), was implanted into the center of the window and a glass coverslip held in place using a circlip to seal the window. Tumors were allowed to establish under the windows and imaging performed 8 – 10 days post-implantation.

**In vivo imaging**

GFP-expressing SCC cancer cells / tumors were implanted under optical windows and imaged 8 – 10 days post-implantation. All images were captured using a custom built multi-modal microscope equipped with an Olympus XLPlan N 25x 1.05 N.A. water immersion lens. Animals were anesthetized using an isofluorane / oxygen mixture and secured on the microscope using a custom built imaging box. Typically images were acquired using at-sample laser powers in the range of 30 – 60 mW for both the pump and Stokes beams, pixel dwell times of 10 𝜇s used with images gathered every 2.6 seconds. Emission signals were spectrally separated using the filter sets detailed in the ‘Microscope Design and Construction’ section.

**Quantification of RBC velocity and hematocrit *in vivo***

Analysis of RBC velocity from blood vessels in which the RBC image appeared undistorted i.e. those moving slower than the frame rate, was performed using manual tracking by utilizing the spot detection feature in Imaris (Bitplane). Multiple RBCs from within a single vessel were tracked in this manner and the mean velocity and standard deviation calculated. For blood vessels in which the RBCs were observed to move faster than the acquisition frame rate i.e. those in which the RBC image appeared smeared, a method adapted from Kamoun et al ^33^ was used. Briefly, the orientation of the scanning mirrors was altered so as the y-scanning direction was parallel to the flowing RBCs within the blood vessel, and the acquisition parameters (resolution, pixel dwell time, image size) adjusted so as to produce elongated RBC tracks within the image. Continuous scanning was performed over a number of frames to acquire multiple images from each vessel. Using ImageJ, tracks with a displacement length greater than 28 μm were traced with the NeuronJ plugin ^43^ and the distance travelled used to calculate the velocity.

Utilizing images acquired from blood vessels in which RBC velocity was deemed to be slow, we used ImageJ to create a mask over the vessel area and then apply a threshold over the RBCs within the vessel. The thresholded area within the mask was then used to calculate the percentage of the masked area occupied by RBCs as a measure of hematocrit. This analysis was performed for each image of a 40 image time-series and the average hematocrit for each vessel calculated.

**Supplementary Figures:**

**Supplementary Figure 1 |** (**a**) Continuous time-lapse imaging shows no sign of visible photo-damage. Representative image series of liver blood flow from a continuous time-lapse of 300 images over 13.5 minutes. Images were acquired with laser powers of 40 mW for the pump and 30 mW for the Stokes beam measured after the objective, through a liver imaging window at a speed of 8 μs / pixel, 2.1 seconds / frame continuously with no signs of visible photo-damage. Cyan, CARS imaging at 2,930 cm^-1^; Red, SHG. (**b**) Single reslice of ex vivo imaged kidney using CARS imaging at 2,845 cm^-1^

**Supplementary Table 1 |** Component List. List of components required to build the optical path used to connect the laser source into the confocal scanning microscope. In addition the filter details used for image acquisition are provided alongside details of the confocal microscope and laser system used. It should be noted that in some cases similar components are available from alternative suppliers.

**Supplementary Movie 1 |** 3D Render of Z-section from Fig. 3a. GFP-labeled SCC cancer cells (Green), collagen matrix imaged using SHG (blue), and a range of cell types including red blood cells (RBC), endothelial cells, and tumor infiltrating immune and stromal cells imaged using CARS at 2,930 cm^-1^ (Red).

**Supplementary Movie 2 |** 3D Render of Z-section from Fig. 3c. GFP-labeled SCC cancer cells (Red) are seen in the collagen network (Cyan) as they invade out from the tumor margin.

**Supplementary Movies 3, 4, 5 and 6 |** RBC velocity *in vivo*. GFP-labeled SCC cancer cells (Green) and a range of cell types including red blood cells (RBC), endothelial cells, and tumor infiltrating immune and stromal cells imaged using CARS (Red). Movies 4 and 5 were recorded using 30 mW pump and 25 mW Stokes power with a speed of 4 𝜇s / pixel, 1.1 seconds / frame continuously, whilst Movies 3 and 6 were recorded using 30 mW pump and 35 mW Stokes power with a speed of 8 𝜇s / pixel, 2.1 seconds / frame continuously.

**Supplementary Movie 6 |** Continuous time-lapse imaging shows no sign of visible photo-damage. Representative time-lapse movie of liver blood flow from a continuous time-lapse of 300 images over 13.5 minutes. Images were acquired with laser powers of 40 mW for the pump and 30 mW for the Stokes beam measured after the objective, acquired through a liver imaging window at a speed of 8 μs / pixel, 2.1 seconds / frame continuously with no signs of visible photo-damage. Cyan, CARS at 2,930 cm^-1^; Red, SHG.
