# Supplementary figures and images for: In vivo imaging of the tumor and its associated microenvironment using combined CARS / 2-photon microscopy
Source: Intravital. 2015 Jun 8;4(1):e1055430. doi: 10.1080/21659087.2015.1055430 (PMC5226011; doi:10.1080/21659087.2015.1055430)

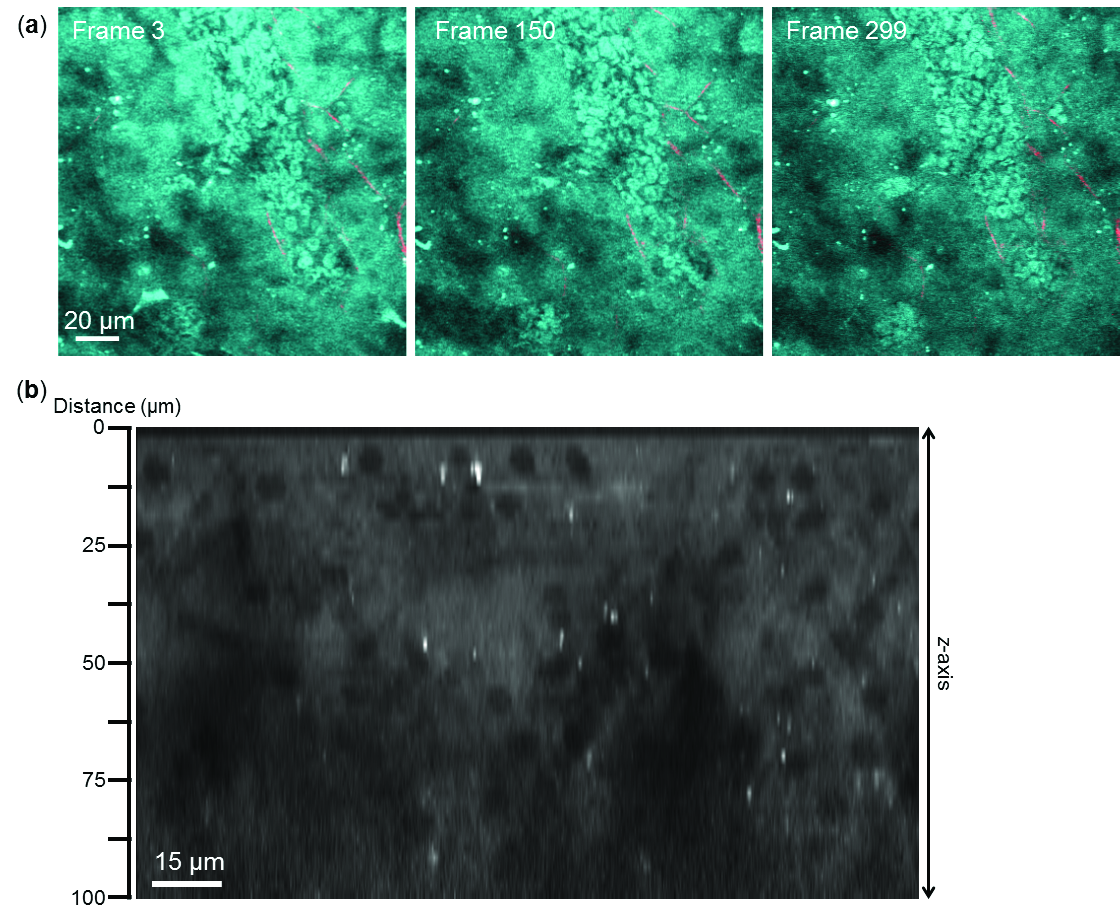

Supplement: Supplementary_Materials.zip [file kinv-04-01-1055430-s007.zip › Figure S1.tif]
